# Protocol to visualize distinct motoneuron pools in adult zebrafish via injection of retrograde tracers

**DOI:** 10.1016/j.xpro.2022.101868

**Published:** 2022-12-10

**Authors:** Maria Bertuzzi, Irene Pallucchi, Abdeljabbar El Manira

**Affiliations:** 1 Department of Neuroscience, Karolinska Institutet, 171 77 Stockholm, Sweden

**Keywords:** Microscopy, Model Organisms, Molecular/Chemical Probes

## Abstract

In adult zebrafish, slow, intermediate, and fast muscle fibers occupy distinct regions of the axial muscle, allowing the use of retrograde tracers for selective targeting of the motoneurons (MNs) innervating them. Here, we describe a protocol to label distinct MN pools and tissue processing for visualization with confocal laser microscopy. We outline the different steps for selective labeling of primary and secondary MNs together with spinal cord fixation, isolation, mounting, and imaging.

For complete details on the use and execution of this protocol, please refer to Pallucchi et al. (2022)^1^ and Ampatzis et al. (2013).^2^

## Highlights

• Protocol to visualize distinct motoneuron pools in adult zebrafish
• Selective labeling of primary and secondary motoneurons with dextran dyes
• Spinal cord fixation, isolation, mounting, and confocal microscopy

## Abstract (editor highlights)

Publisher’s note: Undertaking any experimental protocol requires adherence to local institutional guidelines for laboratory safety and ethics.

## Graphical abstract

**Figure:**
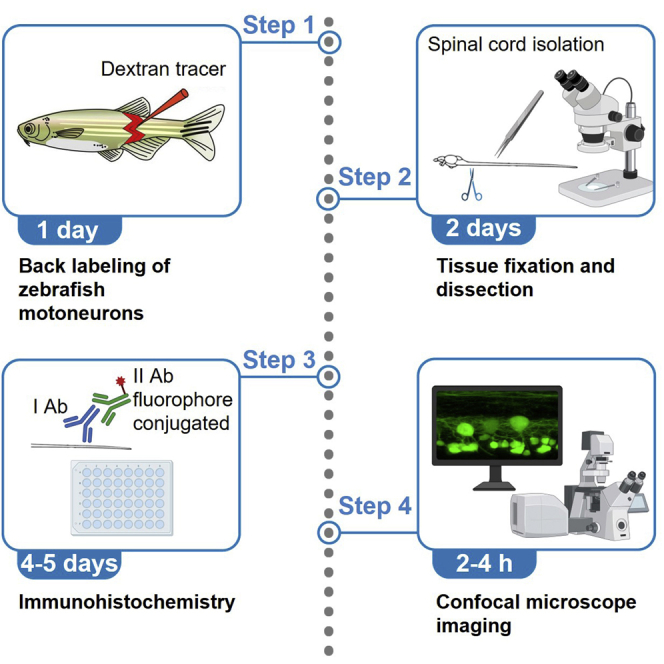

## Before you begin

In zebrafish, motoneurons can be divided into two main types, primary and secondary, depending on the time of their birth, soma size and position in the spinal cord. Primary motoneurons (pMN) are born first, they have large cell bodies and axons, and occupy a dorsal position in the motor column. Secondary motoneurons (sMN) are born later, they have comparatively smaller cell bodies and axons and reside in the ventral part of the motor column. pMNs are fast and innervate the large white muscles. In adult zebrafish, sMNs are heterogenous and comprise three functional subtypes each selectively innervating either fast, intermediate or slow muscles. This classification results in four distinct motoneuron pools.^2^^,^^3^^,^^4^^,^^5^^,^^6^ These pools are organized somatotopically with a defined location in the motor column that relates to the muscle type they innervate. Slow motoneurons are located ventro-laterally, intermediate motoneurons have a ventro-medial location and fast motoneurons are located medio-dorsally in the spinal motor column.

Zebrafish transgenic lines labeling MNs are available, but these do not discriminate among the different MN types due to the lack of specific genetic markers for slow, intermediate and fast MNs. For the analysis of motor circuits, it is necessary to identify and access each MN pool separately, this can only be done through retrograde labeling from specific muscles.

In zebrafish, fast, intermediate and slow muscles are spatially segregated, in contrast to mammals where they are intermingled. The zebrafish slow red muscle fibers occupy a thin lateral strip, the intermediate (or pink) form a wedge-shaped area around the horizontal septum, and the white fast fibers represent the large medial portion of the myotome.^7^^,^^8^^,^^9^ The spatial segregation of muscles fibers in adult zebrafish allows for precise targeting of each muscle type by injecting dextran tracers to retrogradely label the MNs innervating them.^2^^,^^3^^,^^4^^,^^6^ Single or multiple MN pools can be labeled simultaneously over several segments in a single fish by targeting dye injections to the muscle they innervate. The specific labeling of the different MN pools enables their identification and allows for studying their activity, the synaptic input they receive, their morphology and their transmitter phenotypes using immunohistochemical protocols.^1^^,^^2^

### Institutional permissions

Zebrafish (*Danio rerio*) were raised and housed in the Karolinska Institutet, Comparative Medicine Biomedicum (KM-B) animal facility according to established procedures. All experimental procedures followed the EU guidelines and were approved by the Animal Research Ethical Committee in Stockholm. Therefore, researchers must acquire authorization to perform animal work from their relevant institutions before using this protocol.

### Prepare transgenic or wild-type zebrafish

The procedure described here can be used in wild-type or transgenic zebrafish lines. When using a transgenic line with a fluorescent reporter, researchers should use a retrograde dye for the labeling of motoneurons that will not interfere with the fluorophores present in the line.

Raise zebrafish (*Danio rerio*) in the animal facility according to established procedures. Adult animals of either sex can be used, and we recommend using 8–12 week old fish (length: ∼15–20 mm) because the fish is big enough to distinguish the muscle fibers, and the spinal cord is not too thick for the whole-mount confocal imaging.

### Prepare anesthetic stock solution

Prepare the ethyl 3-aminobenzoate methanesulfonate (MS-222) stock solution, 0.3% (w/v).

### Prepare injection pin

**Figure 1:**
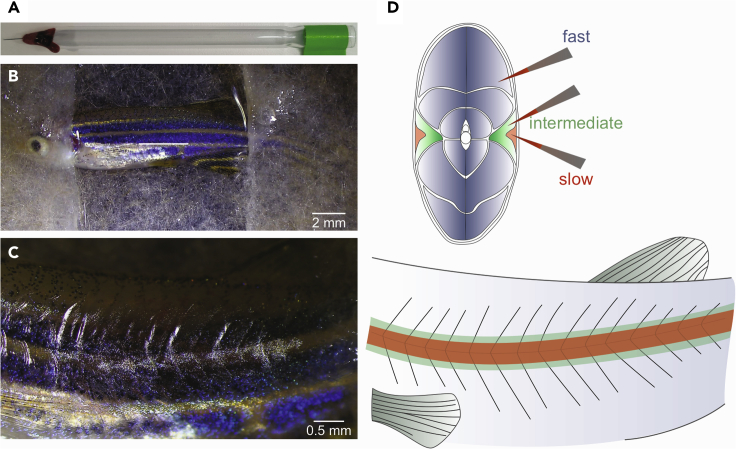
Retrograde labeling of motoneuron pools (A) Injection tool. (B) Fish preparation. (C) Site of injection. (D) Above, schematic showing a coronal section of the muscle fibers in zebrafish (red: slow; green: intermediate; blue: fast) and dye-soaked pins selectively targeting each muscle type. Below, schematic representation of the muscle fibers as seen in (C).

Prepare the injection pipet by embedding a minutien pin (12.5 μm diameter) into a glass Pasteur pipet and seal with wax (Figure 1A). The pin can be sharpened using polishing film for optimal injection with minimal tissue damage.

### Stereo microscope

We use an Olympus SZX10 stereo microscope configured with an ergonomic tilting trinocular viewing head with WHN10×/22 FOV eyepieces. The microscope features a 10:1 zoom range and the dual position nosepiece has DF PLAPO 1× auxiliary lenses, therefore the magnification range is 6.3× - 63×. The focus mount provides coarse and fine focus adjustments.

### Confocal microscope

We use a Zeiss LSM980-Airy2 confocal microscope equipped with a 20× air/dry objective (NA 0.8), a 40× water objective (NA 1.2) and an Airy detector2 for super-resolution capacity with three laser lines (488, 561 and 640).

***Alternatives:*** a fluorescent microscope can be used to visualize labeled motoneurons. Confocal microscopy is not necessary, but it provides a better resolution and image quality.

### Image analysis

We use ZEN (blue edition) from Zeiss to process and analyze the acquired confocal images, but other software can be used.

## Key resources table

**Table:** 

REAGENT or RESOURCE	SOURCE	IDENTIFIER
**Chemicals, peptides, and recombinant proteins**		

Ethyl 3-aminobenzoate methanesulfonate (MS-222)	Sigma-Aldrich	Cat# E10521
Triton X-100	Sigma-Aldrich	Cat# T9284
Bovine serum albumin	Sigma-Aldrich	Cat# A2153
Donkey serum	Thermo Fisher	Cat# D9663
Tetramethylrhodamine-dextran	Thermo Fisher	Cat# D3308
Alexa Fluor 488-dextran	Thermo Fisher	Cat# D22910
Trizma base	Sigma-Aldrich	Cat# T1503
PBS – phosphate-buffered saline (10×) pH 7.4	Invitrogen	Cat# AM9625
Paraformaldehyde 4% in 0.1 M phosphate buffer pH 7.4 (PFA)	Histolab	Cat# HL96753.1000

**Experimental models: Organisms/strains**		

*Danio rerio*: wild-type strain AB. 8–12 week old fish (length: ∼15–20 mm) of either sex	NA	ZFIN ID: ZDB-GENO-960809-7

**Software and algorithms**		

ZEN digital imaging for light microscopy	Zeiss	RRID: SCR_013672

**Other**		

Dumont #5 forceps	Fine Science Tools	Cat# 11295-10
Vannas spring scissors - 2.5 mm cutting edge	Fine Science Tools	Cat# 15000-08
Minutien pins	Fine Science Tools	Cat# 26002-10
Silicon carbide lapping (polishing) sheets	Thorlabs	Cat# LF5P
Olympus SZX10 stereo microscope	Olympus	https://www.olympus-lifescience.com/
Zeiss LSM980-Airy2 confocal microscope	Zeiss	http://www.zeiss.com/microscopy
VECTASHIELD® Antifade Mounting Medium	Vectorlabs	Cat# H-1000-10
48 well plates, TC treated	VWR	Cat# 734-2326

**CRITICAL:** Paraformaldehyde has an acute toxicity by inhalation, ingestion, and dermal exposure. It is both corrosive to the skin and eyes and is a suspected carcinogen. Wear a lab coat, nitrile exam gloves and handle PFA under a ventilated hood.

## Materials and equipment

**Table:** MS-222 stock solution, pH 7.2

Reagent	Final concentration	Amount
Ethyl 3-aminobenzoate methanesulfonate	0.3% (w/v)	0.15 g
ddH_2_O	N/A	Up to 50 mL

Adjust the pH with 1 M Trizma-base pH 9. Store at 4°C. Stable for 1–2 months.

**CRITICAL:** overexposure to MS-222 in humans may cause skin, eye and respiratory irritation. Wear a lab coat, nitrile exam gloves and safety glasses when handling powder.

**Table:** Triton X-100 in PBS stock solution

Reagent	Final concentration	Amount
Triton X-100	0.5% (v/v)	2.5 mL
1× PBS	N/A	Up to 500 mL

Stable for years if stored sealed at room temperature (18°C–25°C).

**Table:** BSA 20% stock solution

Reagent	Final concentration	Amount
Bovine Serum Albumin	20% (w/v)	10 g
ddH_2_O	N/A	Up to 50 mL

Prepare 0.5–1 mL aliquots and store at −20°C. Stable for years. Avoid freeze-thaw cycles and if necessary, store at 4°C between 2–7 days.

### Donkey serum

Prepare 0.5 mL aliquots and store at −20°C. Stable for years.

**Table:** Blocking solution

Reagent	Final concentration	Amount
BSA 20% stock solution	1% (v/v)	50 μL
Donkey serum	4% (v/v)	40 μL
Triton X-100 in PBS stock solution	N/A	Up to 1 mL

To be prepared and used fresh.

### Primary and secondary antibody mix

Prepare the antibodies mix in Triton X-100 in PBS following the manufacturer’s recommended dilutions. To be prepared and used fresh.

## Step-by-step method details

The whole protocol must be performed within 10 days to ensure the best staining. The fluorescence should be detectable at least for a month, but it loses intensity over time because dextran dyes degrade.

### Back labeling of motoneurons

**Timing: 16–24 h (for step 1)**

This section describes how to retrogradely label motoneurons by injecting dextran dyes into zebrafish muscles.

1. Prepare the injection pin.
  a. Place some crystals (approx. 0.1 mg) of the fluorescent dextran dye on a clean glass slide.
  b. Add 2 μL of distilled water to the dye, mix well and then let the mix dry for some minutes.
  c. Dip the tungsten pin when the dye mix has the consistency of a paste. Avoid letting the mix desiccate and add some microliters of water if needed.
2. Prepare the fish (Methods video S1).
  a. Prepare the anesthetic solution by diluting the MS-222 stock solution to a final concentration of 0.03% in fish tank water.
  b. Place the fish in the anesthetic solution for about 30–60 s until deep anesthesia is reached.
    ***Note:*** In zebrafish, deep anesthesia is characterized by a lack of movement, few or no opercular movement, and a lack of response to a soft touch.
  c. Place the fish laterally on a wet paper tissue under a stereo microscope.
  d. Secure the fish position by covering the head and the tail with similar paper tissue (Figure 1B).
  Methods video S1. Preparation of zebrafish for dextran injection, related to step 2
3. Inject the dye in selective muscle fibers (Methods video S2).
  a. Dry the surface of the fish and scrape away a few scales using forceps to gain better access to the targeted muscle.
  b. Inject the dye-soaked pin in the target muscle fibers (Figures 1C and 1D). If needed, soak the pins in the dye multiple times and repeat the injection.
  c. Let the fish recover from anesthesia in fresh fish tank water.
  d. Keep the animal in a regular fish tank for at least 2 h or overnight (14–20 h) to allow for retrograde transport of the tracer.
4. Repeat the steps 1–3 on the desired number of fish depending on the experimental design.

**CRITICAL:** Commonly used dyes are rhodamine-dextran 3,000 MW (Thermo Fisher) or Alexa Fluor 488-dextran 10,000 MW (Thermo Fisher). Dextran dyes are retrogradely transported from the severed axon to the cell soma and the speed of their transport is related to their MW. Both 3,000 and 10,000 MW dextrans reach the MN soma after less than 2 h.

**CRITICAL:** Make sure to carry out the back labeling procedure within 5–10 min, since prolonged anesthesia may be lethal for the animal. For this reason, it is recommended to carry out the injections on one fish at a time. To help the recovery, fish water can be kept at a temperature of 26°C–28°C.

Methods video S2. Injection of dextran rhodamine in anesthetized zebrafish, related to step 3

### Tissue fixation and spinal cord isolation

**Timing: 2 days (for step 5)**

This section describes how to fix and isolate zebrafish spinal cord.

**Figure 2:**
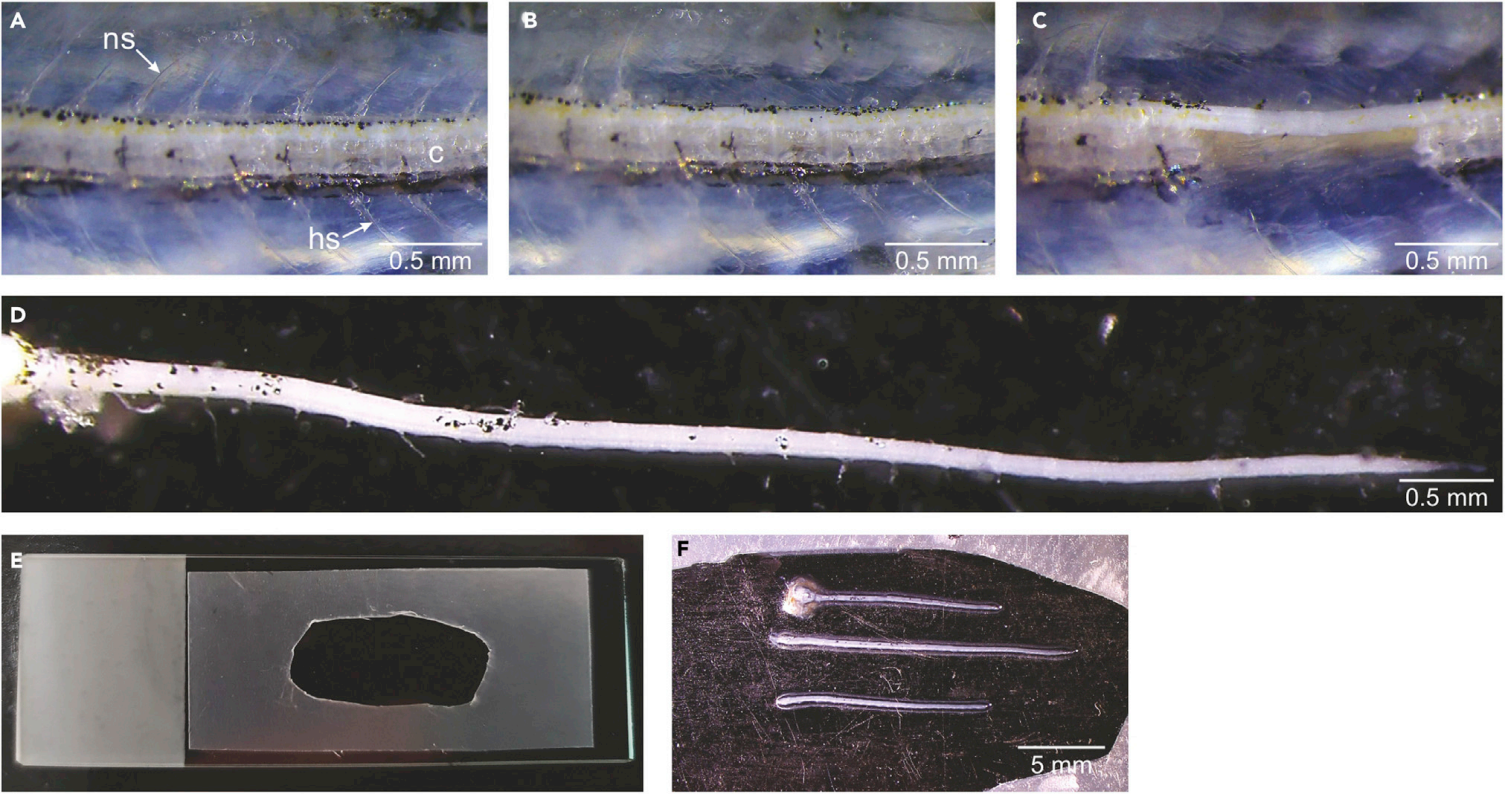
Spinal cord dissection (A–C) Sequential phases of the dissection of zebrafish spinal cord (ns: neural spine, hs: hemal spine, c: centrum). (D) Isolated spinal cord positioned laterally. (E) Glass slide prepared with a parafilm frame. (F) Example showing three spinal cords positioned on a glass slide.
  e. Discard the 1× PBS and add 4% PFA in the dish and keep it at 4°C 2–24 h to allow for optimal fixation.
6. Spinal cord isolation.
  a. Discard the 4% PFA, rinse once with 1× PBS and fill the dish with 1× PBS.
  b. Perform the spinal cord isolation using forceps under a stereomicroscope.
    i. Remove the neural spines (ns) and vertebrae arches to facilitate the dissection (Figure 2B).
    ii. Remove the hemal spines (hs) and the centrum (c) to expose the spinal cord (Figure 2C).

***Note:*** Once the vertebrae are removed (Figure 2D), the spinal cord can be transferred to a multiwell plate filled with 1× PBS and stored at 4°C until further processing. It is recommended to perform the next steps within 48 h to avoid degradation of dextran dyes.

5. Tissue fixation.
  a. Deeply anesthetize all the injected fish with 0.1% MS-222 solution.
    ***Note:*** High MS-222 concentration will keep the fish under deep anesthesia during dissection.
  b. Place the fish on a paper towel for a few seconds to remove the excess of water.
  c. Glue the fish laterally on a glass Petri dish using superglue. Cover the head and the tail with the glue and immediately submerge the fish in 1× PBS.
  d. Using surgical tools, remove internal organs, the skin first and then the muscles to expose the vertebrae (Figure 2A).

### Tissue labeling and mounting

**Timing: 4–5 days (for step 7)**

This section describes the tissue immunolabeling (optional) and the spinal cord mounting on microscope slides.

7. Optional: Immunohistochemistry.
  Researchers can combine retrograde labeling of MNs with immunohistochemistry, which can be carried out in a multiwell plate (48-well for example) as follows.
  a. Wash the tissue extensively with 1× PBS (3 × 15 min).
  b. Block non-specific binding sites with blocking solution for 30 minutes at room temperature (18°C–25°C).
  c. Incubate in primary antibody mix at 4°C for the appropriate amount of time. Wash extensively with 1× PBS (3 × 15 min).
    ***Note:*** Primary antibody incubation is variable. Overnight (14–20 h) is enough for highly expressed proteins (like reporters), but a range of 36–96 h works better for endogenous proteins.
  d. Incubate with secondary antibody mix at 4°C overnight (14–20 h). Wash extensively with 1× PBS (3 × 15 min).
    ***Note:*** The secondary antibody incubation can be shortened to 2–6 h at room temperature (18°C–25°C); however, this is not recommended.
8. Tissue mounting.
  a. Cut a piece of parafilm to fit the microscope glass slide and cut out the middle of the parafilm where the spinal cord will be placed.
  b. Press the parafilm on the slide and warm it up gently on a heating plate to avoid that it detaches from the glass.
    ***Note:*** The parafilm frame on the glass slide is necessary to preserve the spinal cord shape and avoid compression of the tissue.
  c. Let the slide cool down before placing the spinal cord (Figure 2E).
  d. Position gently the spinal cord in the desired orientation on the slide using forceps (Figure 2F).
  e. Add non-hardening mounting media. Coverslip the sample.
    ***Note:*** While most microscope mounting media are suitable, hardening media are not recommended because they will alter the shape of the tissue.

### Confocal microscopy

**Timing: 1–4 h (for step 9)**

This section describe the image acquisition process at the confocal microscope.

**Figure 3:**
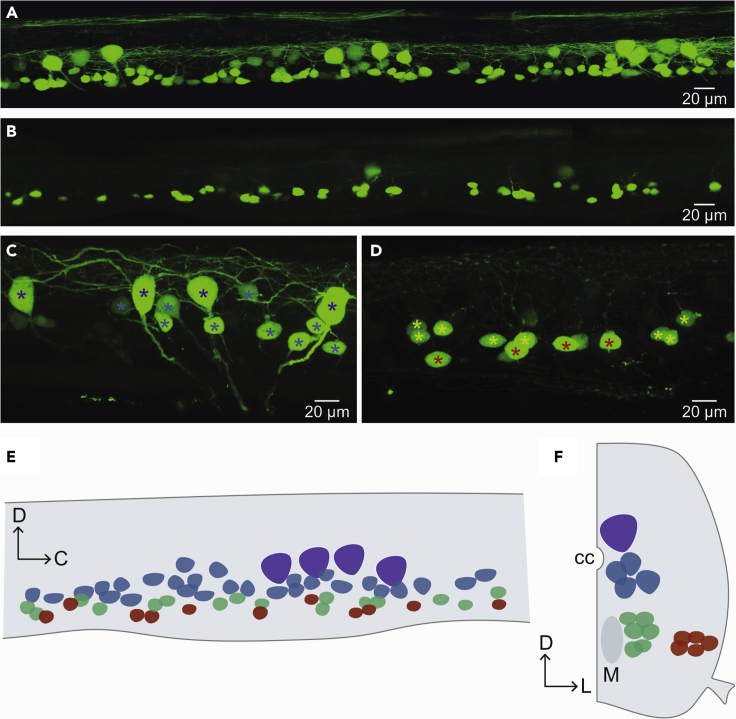
Confocal microscopy imaging (A) Confocal image showing a lateral view of three segments with primary and fast secondary motoneurons. (B) Confocal image showing slow and intermediate motoneuron labeling in three subsequent spinal cord segments. (C) High magnification image showing primary (dark blue asterisks) and fast secondary (light blue asterisks) MNs. (D) High magnification image of slow (red asterisks) and intermediate (green asterisks) MNs. (E) Schematic representation of a spinal cord hemisegment in a lateral view, showing the position of the different MN pools (dark blue: fast pMNs; light blue: fast sMNs; green: intermediate sMNs; red: slow sMNs). (F) Schematic of a coronal view of one side of the spinal cord showing the position of the different motoneuron pools (colors are as in E). M: Mauthner axon, cc: central canal.

9. Acquisition of confocal images.
  a. Prepare the Zeiss LSM980-Airy2 confocal microscope equipped with a 20× (NA 1.1) objective.
  b. Focus on the spinal cord using the fluorescence or the bright field.
  c. Move to the acquisition mode and set up the desired channels.
  d. Acquire the part of the spinal cord where MNs were back labeled. If the protocol has been successful, images as those shown in Figure 3A should be obtained.

## Expected outcomes

Selective injection of specific muscle fiber types should lead to labeling of distinct motoneuron pools. Fast primary MNs have large somata, considerable dendritic arborization and are located in the medio-dorsal portion of the spinal cord. There are 4 pMNs per hemisegment (Figures 3A, 3C, 3E, and 3F).

Fast secondary MNs have relatively large somata, extensive dendrites and occupy the medio-dorsal portion of the spinal cord (Figures 3A, 3C, 3E, and 3F). Intermediate secondary MNs are located medio-ventrally, they have smaller somata, and their dendritic arborization is less extensive (Figures 3B and 3D–3F). Slow secondary MNs are located ventro-laterally, they have soma size and dendritic arborization comparable to intermediate MNs (Figures 3B and 3D–3F).

The procedure described here allows to gain access to distinct MN pools innervating specific muscle types. This enables comparing their physiological, morphological and transmitter features.^10^ The retrograde labeling of MNs also affords their electrophysiological characterization in live tissue. This procedure combined with electrophysiological recordings allows for gaining detailed information on the intrinsic properties of the different motoneuron pools, their connectivity with premotor interneurons as well as the extent of their dendritic arborizations. In addition, this procedure can be used in mutant or transgenic animals modeling motoneuron diseases.

## Limitations

In general, it is difficult to label all motoneurons innervating a given muscle type and therefore multiple injections should be performed at different segments and repeated in different animals. Moreover, MN staining intensity is proportional to the quantity of dye incorporated. MNs with larger axons will be strongly labeled. This protocol is applicable only to juvenile-adult zebrafish, as larvae have different muscle composition and the pins used in this study are not suitable for injections in larvae.

## Troubleshooting

### Problem 1

Zebrafish do not recover from anesthesia after back labeling of motoneurons (related to steps 1-2-3).

### Potential solution

• Adjust the anesthetic concentration. Make sure to prepare fresh MS-222 at the right concentration. The solution can be stored at 4°C for at least one month but should be reprepared as necessary.
• Do not keep zebrafish under deep anesthesia for more than 5–10 min.
• Make sure you inject the dye only in the muscle to avoid organ lesions.

### Problem 2

Muscle fibers are not easily distinguishable (related to step 3).

### Potential solution

Please use Figure 1 as a reference and gently remove some scales to expose the muscle fibers. Use the recommended size and age of zebrafish to facilitate the identification of the different muscle fiber types. Illumination at an appropriate angle usually helps identify the horizontal septum.

### Problem 3

Unsuccessful spinal cord isolation (related to steps 5 and 6).

### Potential solution

The spinal cord may break during the dissection, so researchers should make sure that the tissue has been properly fixed. Generally, a 2-h fixation with 4% PFA is enough, but it is recommended to use a longer fixation to facilitate the dissection. The procedure requires some training for achieving optimal results.

### Problem 4

No or very few motoneurons are visible (related to step 9).

### Potential solution

The concentration of the dye is important when injecting it into muscles. Too low concentration results in a low fluorescent signal and too high concentration could damage MNs. Tissue should also be processed according to the time scale described because dextran dyes deteriorate over time.

### Problem 5

Unsuccessful immunohistochemistry (related to steps 7–9).

### Potential solution

• Adjust the concentration of antibodies according to the manufacturer’s recommendation.
• Increase the incubation time since whole spinal cord requires a longer time for reaching optimal antibody penetration. For primary antibodies, overnight incubation (14–20 h) is enough for highly expressed proteins (like reporters), but a range of 36–96 h works better for endogenous proteins. For secondary antibodies, overnight incubation (14–20 h) is sufficient.
• Some primary antibodies may require a short tissue fixation in order to bind to the antigens. In this case, perform a 2-h spinal cord fixation with 4% PFA. Alternative fixation methods can be used.

## Resource availability

### Lead contact

Further information and requests for resources and reagents should be directed to and will be fulfilled by the lead contact, Abdel El Manira (abdel.elmanira@ki.se).

### Materials availability

This study did not generate new unique reagents.

## Data Availability

This study did not generate/analyze datasets or code.

## References

[1] Pallucchi I., Bertuzzi M., Michel J.C., Miller A.C., El Manira A. (2022). Transformation of an early-established motor circuit during maturation in zebrafish. Cell Rep..

[2] Ampatzis K., Song J., Ausborn J., El Manira A. (2013). Pattern of innervation and recruitment of different classes of motoneurons in adult zebrafish. J. Neurosci..

[3] Ampatzis K., Song J., Ausborn J., El Manira A. (2014). Separate microcircuit modules of distinct v2a interneurons and motoneurons control the speed of locomotion. Neuron.

[4] Gabriel J.P., Ausborn J., Ampatzis K., Mahmood R., Eklöf-Ljunggren E., El Manira A. (2011). Principles governing recruitment of motoneurons during swimming in zebrafish. Nat. Neurosci..

[5] Song J., Ampatzis K., Björnfors E.R., El Manira A. (2016). Motor neurons control locomotor circuit function retrogradely via gap junctions. Nature.

[6] Song J., Dahlberg E., El Manira A. (2018). V2a interneuron diversity tailors spinal circuit organization to control the vigor of locomotor movements. Nat. Commun..

[7] de Graaf F., van Raamsdonk W., van Asselt E., Diegenbach P.C. (1990). Identification of motoneurons in the spinal cord of the zebrafish (Brachydanio rerio), with special reference to motoneurons that innervate intermediate muscle fibers. Anat. Embryol..

[8] van Raamsdonk W., Pool C.W., te Kronnie G. (1978). Differentiation of muscle fiber types in the teleost Brachydanio rerio. Anat. Embryol..

[9] van Raamsdonk W., van't Veer L., Veeken K., Heyting C., Pool C.W. (1982). Differentiation of muscle fiber types in the teleost Brachydanio rerio, the zebrafish. Posthatching development. Anat. Embryol..

[10] Berg E.M., Björnfors E.R., Pallucchi I., Picton L.D., El Manira A. (2018). Principles governing locomotion in vertebrates: lessons from zebrafish. Front. Neural Circuits.

